# Effects of COVID-19 Lockdown on Physical Activity and Dietary Behaviors in Kuwait: A Cross-Sectional Study

**DOI:** 10.3390/nu13072252

**Published:** 2021-06-30

**Authors:** Ahmad Salman, Kennedy Ouma Sigodo, Fatima Al-Ghadban, Badreya Al-Lahou, Maha Alnashmi, Souhail Hermassi, Sungsoo Chun

**Affiliations:** 1 Ministry of Health, Safat 13001, Kuwait; 2 Kuwait Public Policy Centre, General Secretariat of the Supreme Council for Planning and Development, Safat 13001, Kuwait; sungsoo.chun@aucegypt.edu; 3 Physical Education Department, College of Education, Qatar University, Doha 2713, Qatar; shermassi@qu.edu.qa; 4 Department of Public Health, Glasgow Caledonian University London, London E1 6PX, UK; Kennedy.Sigodo@gcu.ac.uk; 5 College of Public Health and Human Sciences, Oregon State University, Corvallis, OR 97331, USA; alghadbf@oregonstate.edu; 6 Environment and Life Sciences Research Center, Kuwait Institute for Scientific Research, Safat 13109, Kuwait; blahou@kisr.edu.kw; 7 Health Informatics and Information Management Department, Faculty of Allied Health Sciences, Kuwait University, Safat 13110, Kuwait; maha.alnashmi@ku.edu.kw; 8 The Institute of Global Health and Human Ecology, The American University in Cairo, AUC Avenue, New Cairo 11835, Egypt

**Keywords:** COVID-19, physical activity, dietary behavior, Kuwait

## Abstract

The Coronavirus disease (COVID-19) pandemic has brought about drastic measures that have significantly altered the norms of daily living. These measures have affected human behaviors in disparate ways. This study seeks to understand the impact of the pandemic on physical activity and dietary behavior among adults living in Kuwait. A cross-sectional survey was conducted between 18 June and 15 July 2020, using a questionnaire disseminated through social media, including WhatsApp and Facebook. The target population was individuals aged 21 years or older living in the State of Kuwait. The study included 679 respondents; 57.9% were females, and 67.7% were Kuwaiti nationals. Both genders reported an increased consumption of vegetables, fruits, and carbohydrates, and a decreased consumption of fish and sugary drinks. Compared to males, females reported eating more during the outbreak than their pre-pandemic eating behaviors (32.3% vs. 35.9%, *p* < 0.05). Approximately one-third of respondents (33.1%) reported performing less than 30 min of physical activity or exercise in a week, and 36.4% of respondents rated their quality of sleep as ‘poor’ or ‘very poor’. The rate of smoking cigarettes among males was significantly higher than in females (40.6% vs. 5.3%, *p* < 0.001). Physical activity was positively correlated with vegetable consumption and quality of sleep. Quality of sleep was negatively correlated with the consumption of sweets and snacks, just as the consumption of vegetables was negatively correlated with the consumption of sugary drinks. The overall negative impact of the COVID-19 pandemic in Kuwait necessitates the development of health promotion interventions to support positive physical activity and dietary behaviors using alternative coping strategies among the residents of Kuwait.

## 1. Introduction

After Coronavirus disease (COVID-19) was confirmed at the end of December 2019 in Wuhan city, Hubei Province, China, the virus rapidly spread to become a public health emergency of international concern by 30 January 2020, and was declared a global pandemic on 15 March 2020 [1]. Since then, the disease has caused more than 3.1 million deaths and multiple morbidities that continue to increase globally [2]. Furthermore, the disruptions caused to all aspects of daily living and the stringent measures necessitated in stemming the spread of the virus have both had an immense impact on peoples’ lives, with numerous health and socioeconomic consequences. Since Kuwait’s first case of COVID-19 was confirmed on 24 February 2020, the State of Kuwait has faced profound public health challenges due to changes in policies and procedures related to controlling and mitigating the spread of COVID-19 [3]. In addition, Kuwait has a unique demographic and social structure. For instance, there are strict gender roles where females are primarily responsible for household chores. Moreover, it is a very social society in which multiple generations live in the same household. Therefore, the COVID-19 lockdown imposed in Kuwait may impact the health and well-being of its population, which differs from experiences in other countries employing similar lockdown measures to combat COVID-19.

The COVID-19 pandemic in Kuwait has brought many changes to people’s behavior. The COVID-19 outbreak has altered life patterns and the perception of infectious diseases among the general public. Many studies on behaviors and perceptions of COVID-19 provide an insight into how people view and react to the disease in the face of the outbreak [4,5,6,7]. There have been reports of changes in peoples’ diet, physical activity, and smoking and alcohol consumption [4,7]. While some changes have been welcomed due to their propensity to promote health and reduce the risk of severe disease, others have been maladaptive and deemed as imminent threats against the pandemic and multiple public health gains in various countries [5,6].

Rapid changes in both the social environment and peoples’ behaviors have negatively impacted well-being [8]. Physical inactivity, smoking, and obesity are among the risk factors of non-communicable diseases (NCDs) in Kuwait [9,10,11,12]. Kuwait has the highest prevalence of insufficient physical activity globally (around 65% of adults do not meet sufficient levels of physical activity) [9]. Hence, any negative implications on diet and physical activity from the pandemic-related disruptions are bound to aggravate the already dire situation.

It is against this background that we identified the imperative need for a comprehensive evaluation of the changes in dietary behavior and physical activity among Kuwaiti residents. The knowledge of the changes in these modifiable risk factors among the Kuwaiti public is important for the rapid development of public health intervention programs to mitigate any potential negative health consequences [9,13]. Moreover, this study unveils the nature and prevalence of the effects of a pandemic such as COVID-19 on health behaviors, and provides recommendations for future preparedness. This study sought to understand the pandemic’s impact on physical activity and dietary behavior among the residents of Kuwait.

## 2. Materials and Methods

### 2.1. Survey Method and Target Population

This study was designed to collect cross-sectional data using online surveys during the initial government-mandated COVID-19 containment strategies (June/July 2020) in Kuwait. The survey applied a non-random sampling method. The questionnaire was developed using Microsoft Forms and disseminated through social media platforms, including WhatsApp and Facebook, using a sharable link to the questionnaire. Responses were collected from 19 June to 15 July 2020. Consent was obtained from volunteer participants using the first section of the questionnaire, which identified the survey’s purpose and provided information on ethical considerations. This section also included screening questions that confirmed inclusion criteria of being 21 years of age or over and living in the State of Kuwait, and exclusion criteria of being younger than 21 years or residing outside of the territory of Kuwait although they are Kuwait citizens. Those who were previously infected with the COVID-19 were included. There were no significant differences in perceived barriers and severity of the COVID-19 between those who were not infected and those who were previously infected with the virus. The total number of respondents was 679 during the survey period.

### 2.2. Questionnaire Design and Tools

A 65-item questionnaire was used to assess health behaviors in various areas, including demographic information, COVID-19 related perceptions and behaviors, dietary and food consumption behaviors, physical activity and exercise behaviors, and related health behaviors. A small pilot study was conducted on a few participants to assess the acceptability and suitability of the e-survey prior to dissemination. All measures were assessed according to two time points, the first being the pre-COVID-19 restriction period, and the initial during COVID-19 restriction period that occurred from 22 March 2020 to 30 August 2020. For the comparison of behavioral differences, the behaviors of participants both the pre- and during COVID-19 restrictions were asked. In this study, the terminology “pre-COVID-19 restrictions” was defined as “before the period that preventing restriction of the COVID-19 spread” while the meaning of “during COVID-19 restrictions” was defined as “from the day that the first COVID-19 restriction was implemented to the day that the respondent was answering the questionnaire”.

#### 2.2.1. Background Information

Respondents self-reported demographic information, including age, gender, nationality, marital status, and education level. Current health conditions such as high blood pressure, diabetes and cardiovascular disease were also self-reported.

#### 2.2.2. Dietary Behavior and Food Consumption

Dietary behavior was assessed using questions related to food consumption and were classified according to the food consumption types recommended for the Middle Eastern food consumption pattern survey [14] and categorized by 10, dividing into two more categories on the Saudi Food Frequency Questionnaire [15] considering the nutritional recommendations during COVID-19 quarantine [16]. The Saudi Food Frequency questionnaire has been validated, with the test-retest reliability of the completed questionnaire being 0.78, indicating a high reproducibility of the questionnaire [15]. Dietary behavior was measured through an assessment of the consumption of ten different food categories both pre- and during COVID-19 restrictions (i.e., carbohydrates, animal products, milk products, fishes, vegetable proteins, vegetable unsaturated fatty acids, fruits, vegetables, sugary drinks, and sweets and snacks), with responses based on a 5-item Likert scale ranging from ‘I eat much more’ to ‘I eat much less’ compared to pre-pandemic times. Two additional items were used to assess the overall perception of changes in dietary behavior and weight status during the COVID-19 outbreak.

#### 2.2.3. Physical Activity and Exercise

Physical activity was defined according to the meeting of the recommended ≥150 min of moderately- to vigorously-intense physical activity per week [17]. Physical activity and exercise behaviors were measured using the International Physical Activity Questionnaire: Short Form (IPAQ-SF) [18]. Respondents self-reported their exercise behavior before and during initial COVID-19 restrictions based on one of the following statements from the Stages of Change scale: (i) Yes, I exercise more regularly than before; (ii) No, I exercise more irregularly than before; (iii) I exercise at the same regularity as I do before; or (iv) I do not exercise.

#### 2.2.4. Perceptions about the COVID-19 Pandemic

For measuring perceptions related to the COVID-19 pandemic, four questions were chosen from a previous study to assess perceived barriers, and two questions were developed to assess self-efficacy [19]. Self-efficacy is the belief and the level of confidence one has related to performing a specific behavior [20,21], and an appropriate level of self-efficacy is helpful in overcoming barriers [22]. Self-efficacy was indicated by the respondent’s sense of control over preventing and avoiding COVID-19. Perceived barriers and self-efficacy items on the survey relate to following public health recommendations, including handwashing and mask-wearing.

### 2.3. Research Ethics

This research was conducted in accordance with the Declaration of Helsinki. Ethical approval was provided by the Research Ethics Review Committee of the Kuwait Ministry of Health on 8 June 2020 (#1487). This study adhered to the Strengthening the Reporting of Observational Studies in Epidemiology (STROBE) guidelines [23].

### 2.4. Method of Analysis

Characteristics of the study population were described using proportions for categorical variables and means, and standard deviations for continuous variables. Data were checked for the assumptions of normality and homoscedasticity. Cross tabs Chi-square test and independent-samples t-test were conducted to identify gender differences in variables related to physical activity and dietary behavior patterns as well as other health behaviors. Pearson’s correlation analysis was performed to identify intercorrelation between dependent and independent variables. All statistical analyses were conducted using IBM Statistical Package and Services Solutions (SPSS) software version 25 (IBM Corp, Armonk, New York, NY, USA), and assessed at a *p*-value < 0.05 for statistical significance.

## 3. Results

### 3.1. General Characteristics

Among 679 respondents, 57.9% were female and 67.7% were Kuwaitis. Age group categories were represented relatively evenly, with 28.7% of respondents in their 20s, 29.3% in their 30s, 23.0% in their 40s, and 19.0% in the age range of 50 years or above. The average age was 37.7 (SD: 11.6). Of the total sample, 63.8% were married, and 70.3% reported having a college-level education or higher. About two-thirds (66.9%) of the respondents reported not having any diseases or health conditions.

### 3.2. Dietary Behaviors

Table 1 reports on dietary behaviors comparing food consumption before and during the COVID-19 outbreak, stratified by gender. In the overall sample, adults reported eating more vegetables (+0.29), fruits (+0.19), and carbohydrates (+0.15), and less fish (−0.46) and sugary beverages (−0.45) during COVID-19 compared to before the pandemic. Significant gender differences in food consumption before and during covid were observed. Males consumed significantly more meats, eggs, and milk products during the pandemic compared to their pre-pandemic consumption. On the other hand, females consumed significantly more sweets and snacks during the pandemic than their pre-pandemic consumption.

### 3.3. Health Behaviors

Table 2 reports on the respondents’ health behaviors during the COVID-19 outbreak, stratified by gender. Over a third of respondents (34. 3%) reported eating more than before the COVID-19 outbreak, and 35.8% thought they had gained weight since March 2020. Female respondents reported eating more during the outbreak compared to male respondents (35.9% vs. 32.3%, *p* < 0.05). About one-third of respondents (33.1%) reported that they did not achieve at least 30 min of physical activity per week, with no differences observed between genders. Moreover, 36.4% of the respondents reported that their quality of sleep was ‘poor’ or ‘very poor’, although more females reported sleeping five or less than five hours per day compared to males (12.5% vs. 10.1%, *p* < 0.05). The rate of smoking cigarettes among males was significantly higher than in females (40.6% vs. 5.3%, *p* < 0.001). About 15% of males reported that they increased their amount of smoking during the COVID-19 outbreak compared to before the pandemic.

Looking at the correlations between health behaviors (Table 3), each health behavior was significantly correlated with other health behaviors, except for smoking shisha. Days of physical activity were positively correlated with ‘quality of sleep’ and vegetable consumption, and negatively correlated with ‘consumption of sugary drinks’ and ‘consumption of sweets and snacks’. Sleep hours were positively correlated with good quality of sleep. Quality of sleep was negatively correlated with ‘consumption of sugary drinks’ and ‘consumption of sweets and snacks’, and positively correlated with ‘the days of physical activity’. The ‘consumption of vegetables’ was negatively correlated with ‘the consumption of sugary drinks’.

Perception and attitude about COVID-19 were correlated with food consumption (Table 4). Perceived barriers had strong positive correlations with the consumption of carbohydrates, meats, poultry, and sugary drinks, and negative correlations with the consumption of fruits and vegetables. Meanwhile, self-efficacy had negative correlations with the consumption of sugary drinks and sweets and snacks, as well as positive correlation with vegetable consumption.

## 4. Discussion

Before the COVID-19 outbreak, Kuwait (similar to other countries) was trying hard to achieve many targets in the Sustainable Development Goals to reduce mortality rates related to NCDs. Now, the pandemic is making it even more challenging. Apart from being a physical health risk, COVID-19 has a wide-ranging adverse impact on obesity, physical activity, and other health behaviors. These risk factors have a ripple effect in the long run. Therefore, opportunities for scaling up action on NCDs should be taken both immediately and as part of long-term efforts for strengthening health systems. The findings from this study revealed the concerning impact that the COVID-19 pandemic has had, especially on dietary and physical activity behaviors, among individuals living in Kuwait. Results showed that individuals are experiencing pandemic-related changes in their dietary behaviors, as well as in their consumption of specific foods, levels of physical activity, and other health aspects. As a result of the pandemic-imposed restrictions implemented in March 2020, individuals living in Kuwait have experienced significant changes in their health behaviors in such a short period. These significant changes related to dietary and physical activity behaviors may continue to adversely impact the health of individuals as the pandemic persists, putting them at greater health risks in addition to the risk of COVID-19.

The study found a significant increase in the consumption of vegetables, fruits, and carbohydrates among both genders. The parity in the change in consumption experienced among individuals in Kuwait stands in sharp contrast to findings from studies conducted in Spain and the United States [24,25]. Rodríguez-Pérez et al. (2020) found that more males than females increased their fruit and vegetable intake among Spanish adults [24], while Bhutani et al. (2020) found increased fruit and vegetable consumption among American females compared to males [25]. The parity in consumption between men and women living in Kuwait shows a uniform positive change in consumption of fruits and vegetables across the population.

Increased consumption of vegetables and fruits is a laudable shift towards consuming healthy foods. This increase is attributable to most nutritional guidelines encouraging the eating of fruits and vegetables during the pandemic, albeit without convincing evidence associating food with COVID-19 transmission [26]. Evidence of a healthy shift in dietary consumption has been found in a similar study done in Qatar [27] and other different countries worldwide [28,29,30,31,32,33]. However, this shift was not universal, as several countries reported an increase in the consumption of unhealthy foods [34,35] and a decrease in consumption of fresh fruits and vegetables due to COVID-19-related food insecurities [36]. In Kuwait, the increased consumption of carbohydrates, a macronutrient, with increased consumption of micronutrient-rich fruits and vegetables may prevent a deficiency in micronutrient and fiber intakes, which commonly occurs with the increased consumption of macronutrients [37,38]. Micronutrients have physiological, anti-oxidative, and anti-inflammatory properties and their deficiency may lead to an impaired immune system, putting individuals at increased risk of viral infections [39,40].

Multiple reasons have been provided for the favorable modification of diet during the pandemic in Kuwait. Nutrition is a modifiable determinant of chronic diseases such as obesity and cardiovascular diseases that predisposed individuals to severe illness from the SARS-CoV-2 virus [16,41,42]. The presence of this knowledge and the fear of the novel disease might have promoted individuals living in Kuwait to prioritize increasing their consumption of vegetables and fruits to support their immune systems and protect against contracting COVID-19 [43,44]. In the absence of effective treatments, increased consumption of fruits and vegetables was a cheaper and easily accessible preventive method. Other factors such as a decrease in the consumption of fast food; an increased consumption of fresh and homemade foods among Kuwaitis [43]; and working from home [28] have been attributed to healthier shifts in food consumption. Consuming a more balanced, nutrient-dense diet, in the long-run, has the potential of improving physical health outcomes such as controlling blood sugar, lowering cholesterol levels, etc., and consequently resulting in weight loss among children and adults in Kuwait [8,9].

Changes in dietary behavior were not limited to types of food but quantity as well. While 34.2% of respondents in this study reported eating more than in pre-pandemic times, more females than males reported that they eat more than before the pandemic. Overeating during confinement worsens metabolic consequences such as increased insulin resistance, total body fat, abdominal body fat, and inflammatory cytokines developing from the imbalance between calorie intake and expenditure [45]. Despite numerous findings of increased food intake during the pandemic [46], only a few have found variations in quantities of food consumed between males and females [47]. In a study investigating the impact of COVID-19 isolation among University Students, Gallo et al. observed increased energy intake among females but not males [47]. Similar to the explanation provided by Gallo et al., the increased energy intake in females in Kuwait can be attributed to the increased frequency of snacking on high-energy food items observed in this study [47].

Increased eating among females compared to males in Kuwait can be attributed to the gender variations in psychological changes and challenges caused by the pandemic. The ensuing infodemic, social distancing, and disrupted social lives from the pandemic have caused stress, depression, anxiety and mental health problems among people [48,49,50]. Research shows that pandemic-related stress, anxiety and depressive symptoms disproportionately affect more females than males [51,52,53]. To cope with the stress and anxiety, some people resort to increasing consumption of comfort foods such as chocolates, snacks, and sweets [30,33,54]. Therefore, the increased consumption of ‘comfort’ foods and increased eating among Kuwaiti females can be attributed to the disproportionate susceptibility and burden of pandemic-related stress and negative psychological impacts on women and the associated maladaptive coping strategy.

The rate of smoking cigarettes was much higher among males than females, with more males reporting significant increases in smoking during the pandemic. The higher rates of smoking among males, although significantly higher than that of females, can be argued to represent the pre-pandemic rates of smoking. Data collected in Kuwait before the pandemic showed a higher prevalence of smoking among males compared to females [55,56]. Of importance to this study is the significant increase in smoking during the pandemic among males compared to females. A study conducted in Holland found a greater proportion of smokers who reported smoking more compared to those who reported smoking less [57]. Increased smoking was also observed in China [58], Australia [59] and United States [60]. The increase in smoking behavior during the pandemic can be attributed to increased stress [60] and physical inactivity [61]. Variations in coping mechanisms between males and females can be attributed to increased smoking among males and increased eating in females. It can be argued that, while females eat more to cope with stress, males smoke more. Both represent maladaptive coping responses that threaten to eliminate the gains of reducing the prevalence of non-communicable diseases within the Kuwaiti population [9].

The findings show that healthy behaviors and practices are significantly associated with other health-enhancing behaviors and practices. These were evidenced by the strong positive correlation between physical activity, quality of sleep, and vegetable consumption. Moreover, the same association was evident in the negative correlations between physical activity and the consumption of sugary drinks, sweets and snacks. In a chain of association, the emerging health behaviors were further associated with other health-enhancing behaviors. These positively affective processes underlying health behaviors are explained by the upward spiral theory of lifestyle change [62]. According to this theory, a positive feeling perceived from a healthy behavior may trigger long-term adherence to other positive health behaviors. The resultant positive health behaviors and practices, such as increased vegetable consumption, decreased intake of sugary drinks, and increased sleeping hours, that were reported in this study, may be explained as nonconscious behaviors motivated by engaging in physical activity [62]. This finding is of great implication in promoting positive behavior change during the pandemic. It offers the likelihood of directly targeting specific health practices and behaviors that are deemed easily amenable to change, while at the same time indirectly pursuing changes in difficult behaviors. For instance, promoting physical activity to target healthy eating, improve sleep, reduce depressive symptoms, stress, and anxiety, or reduce obesity with the overall reduction in risk to severe COVID-19 outcomes [63].

Finally, the completeness and accuracy of this study’s findings may be limited by memory recall errors that occur when collecting self-reported data using retrospective surveys [64]. Another limitation is that it was not able to ask for detailed dietary supplements (ex, probiotics, vitamins, etc.,) through the cross-sectional survey. In further research, the detailed dietary supplement that the respondents consumed will be beneficial in interpreting the results.

## 5. Conclusions

The results of this survey showed both favorable and unfavorable changes in health behaviors among residents of Kuwait. Favorable health behaviors were marked by increased consumption of fruits and vegetables and decreased consumption of sugary drinks among males and females. Unfavorable changes to health behavior included increased eating among females, and increased smoking among males. This study found positive correlations between positive health behaviors, revealing an opportunity for the utilization of this relationship between healthy behaviors and practices in broader interventions aiming to alleviate the disparate negative impacts of the pandemic on health. The overall negative impact of the COVID-19 pandemic in Kuwait necessitates the development of health promotion interventions to offer alternative healthy coping strategies among the residents of Kuwait. This study provided an opportunity for stakeholders to become better prepared to support people in achieving and maintaining optimal health and well-being, if we were to encounter such events in the future.

## Figures and Tables

**Table 1:** Comparisons of food consumption between before and during the COVID-19 outbreak.

Comparing with the Eating Styling before the COVID-19 Outbreak in Kuwait, Your Current Amount of the Food Consumption Listed Below Are: ^1^	Total (*n* = 679)	Female (*n* = 393)	Male (*n* = 286)	*t*- and (*p*-Value)
	Mean (SD)			
Carbohydrates	+0.15 (0.99)	+0.13 (0.99)	+0.18 (1.00)	−0.661 (0.509)
Meats	−0.06 (0.95)	−0.13 (0.97)	+0.05 (0.93)	−2.535 (0.011) *
Poultry	+0.04 (0.90)	−0.01 (0.89)	+0.10 (0.90)	−1.574 (0.116)
Eggs	+0.04 (0.92)	−0.04 (0.98)	+0.14 (0.93)	−2.470 (0.014) *
Milk products	+0.06 (0.88)	−0.03 (0.87)	+0.18 (0.88)	−2.998 (0.003) *
Fishes	−0.46 (1.05)	−0.51 (1.05)	−0.39 (1.06)	−1.541 (0.124)
Vegetable proteins	−0.13 (0.94)	−0.15 (0.95)	−0.09 (0.93)	−0.844 (0.399)
Vegetable unsaturated fatty acids	−0.09 (1.00)	−0.10 (0.98)	−0.09 (1.03)	−0.152 (0.879)
Fruits	+0.19 (1.02)	+0.16 (1.04)	+0.23 (0.98)	−860 (0.390)
Vegetables	+0.29 (0.95)	+0.27 (0.98)	+0.32 (0.92)	−0.687 (0.490)
Sugary drinks	−0.45 (1.19)	−0.42 (1.20)	−0.50 (1.18)	0.919 (0.359)
Sweets and snacks	0.00 (1.22)	+0.23 (1.24)	−0.16 (1.18)	3.075 (0.002) *

^1^ −2: I eat much less; −1: I eat less; 0: I eat the same as before; 1: I eat more; 2: I eat much more. * *p* < 0.05.

**Table 2:** Health behaviors during the COVID-19 outbreak.

Health Behaviors	Total (*n* = 679)	Female (*n* = 393)	Male (*n* = 286)	*χ^2^*- and (*p*-Value)
	%	%	%	
Overall, I eat more than before the COVID-19 outbreak				
Yes	34.3	35.9	32.2	6.686 (0.035)
No	52.9	49.1	58	
I am not sure	12.8	15	9.8	
Overall, I think I have gained more weight since March 2020.				
Yes	35.8	34.4	37.8	0.839 (0.657)
No	55.5	56.7	53.8	
I am not sure	8.7	8.9	8.4	
In the past week, how many days have you done a total of 30 min or more of physical activity?				
None	33.1	35.4	30.1	4.750 (0.690)
1 day	8.1	6.9	9.8	
2 days	9.9	9.4	10.5	
3 days	13.8	14.2	13.3	
4 days	8.2	8.1	8.4	
5 days	9.7	9.9	9.4	
6 days	4.9	4.1	5.9	
7 days	12.2	12	12.6	
During the COVID-19 outbreak, do you think you are doing exercise more regularly than you do before the COVID-19 outbreak?				
Yes, I exercise more regularly than before	22.8	22.6	23.1	3.107 (0.375)
No, I exercise more irregularly than before	34.3	33.8	35	
I exercise at the same regularity as I do before	11.6	10.2	13.6	
I do not exercise	31.2	33.3	28.3	
How many do you usually smoke a day? (include electric cigarettes)				
None	79.8	94.7	59.4	132.397 (0.000)
5 or less than 5 EA	5	2.3	8.7	
5–9 EA	3.2	1.3	5.9	
10–19 EA	6	1.5	12.2	
Over 20 EA	5.9	0.3	13.6	
How many times do you usually smoke shisha a day?				
None	93.4	94.9	91.3	4.299 (0.231)
Once or twice	4.7	3.3	6.6	
3–4 times	1	1	1	
Over 5 times	0.9	0.8	1	
During the COVID-19 outbreak, do you think you smoke more than you do before the COVID-19 outbreak?				
Yes, I smoke more than before	9.1	4.3	15.7	123.497 (0.000)
No, I do smoke less than before	5	2.8	8	
I smoke the same amount as before	10.3	2	21.7	
I do not smoke	75.6	90.8	54.5	
How many hours do you usually sleep a day? (include nap)				
5 or less than 5 h	11.5	12.5	10.1	9.635 (0.022)
6–7 h	42.3	37.4	49	
8–9 h	35.6	37.9	32.5	
Over 10 h	10.6	12.2	8.4	
How do you think about the quality of your sleep compared to what you did before the COVID-19 outbreak?				
Very poor	12.7	14.5	10.1	5.918 (0.205)
Poor	23.7	24.4	22.7	
Same	32.5	29.5	36.7	
Good	21.6	21.4	20	
Very good	9.4	10.2	8.4	

**Table 3:** Correlations between health behaviors.

	Days of Physical Activity	Daily Amount of Smoking Cigarette	Daily Amount of Smoking Shisha	Sleep Hours	Quality of Sleep	Vegetable Consumption	Sugary Drinks	Sweets and Snacks
Days of physical activity	-							
Daily amount of smoking cigarette	−0.022	-						
Daily amount of smoking shisha	−0.045	0.170 **	-					
Sleep hours	−0.024	0.006	−0.067	-				
Quality of sleep	0.112 **	−0.037	−0.018	0.262 **	-			
Vegetable consumption	0.076 *	−0.050	0.045	0.054	0.028	-		
Sugary drinks	−0.194 **	0.028	0.068	0.083 *	−0.116 **	−0.022	-	
Sweets and snacks	−0.177 **	−0.056	0.014	0.097 *	−0.084 *	−0.078 *	0.597 **	-

*: *p* < 0.05; **: *p* < 0.01.

**Table 4:** Correlations between food consumption, perceived barriers, and self-efficacy.

Food Consumption	Perceived Barriers	Self-Efficacy
Carbohydrates	0.100 **	0.016
Meats	0.124 **	−0.035
Poultry	0.133 **	−0.044
Fruits	−0.095 *	0.075
Vegetables	−0.083 *	0.080 *
Sugary drinks	0.105 **	−0.111 **
Sweets and snacks	0.068	−0.081 *

*: *p* < 0.05; **: *p* < 0.01.

## Data Availability

The raw data supporting the conclusions of this article will be made available by the authors, without undue reservation.
